# Large-Scale Mass Spectrometry Imaging Investigation of Consequences of Cortical Spreading Depression in a Transgenic Mouse Model of Migraine

**DOI:** 10.1007/s13361-015-1136-8

**Published:** 2015-04-16

**Authors:** Ricardo J. Carreira, Reinald Shyti, Benjamin Balluff, Walid M. Abdelmoula, Sandra H. van Heiningen, Rene J. van Zeijl, Jouke Dijkstra, Michel D. Ferrari, Else A. Tolner, Liam A. McDonnell, Arn M. J. M. van den Maagdenberg

**Affiliations:** Center for Proteomics and Metabolomics, Leiden University Medical Center, Einthovenweg 20, 2333 ZC Leiden, The Netherlands; Department of Human Genetics, Leiden University Medical Center, Leiden, The Netherlands; Division of Image Processing, Department of Radiology, Leiden University Medical Center, Leiden, The Netherlands; Department of Neurology, Leiden University Medical Center, Leiden, The Netherlands; Fondazione Pisana per la Scienza ONLUS, Pisa, Italy

**Keywords:** Mass spectrometry imaging, MALDI, Cortical spreading depression, Familial hemiplegic migraine, Preclinical studies

## Abstract

Cortical spreading depression (CSD) is the electrophysiological correlate of migraine aura. Transgenic mice carrying the R192Q missense mutation in the *Cacna1a* gene, which in patients causes familial hemiplegic migraine type 1 (FHM1), exhibit increased propensity to CSD. Herein, mass spectrometry imaging (MSI) was applied for the first time to an animal cohort of transgenic and wild type mice to study the biomolecular changes following CSD in the brain. Ninety-six coronal brain sections from 32 mice were analyzed by MALDI-MSI. All MSI datasets were registered to the Allen Brain Atlas reference atlas of the mouse brain so that the molecular signatures of distinct brain regions could be compared. A number of metabolites and peptides showed substantial changes in the brain associated with CSD. Among those, different mass spectral features showed significant (*t*-test, *P* < 0.05) changes in the cortex, 146 and 377 Da, and in the thalamus, 1820 and 1834 Da, of the CSD-affected hemisphere of FHM1 R192Q mice. Our findings reveal CSD- and genotype-specific molecular changes in the brain of FHM1 transgenic mice that may further our understanding about the role of CSD in migraine pathophysiology. The results also demonstrate the utility of aligning MSI datasets to a common reference atlas for large-scale MSI investigations.

**Electronic supplementary material:**

The online version of this article (doi:10.1007/s13361-015-1136-8) contains supplementary material, which is available to authorized users.

**Graphical Abstract:**
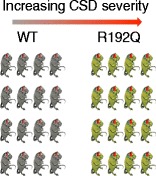
ᅟ

## Introduction

Matrix-assisted laser desorption/ionization (MALDI) mass spectrometry imaging (MSI) is a label-free technique capable of analyzing hundreds of biomolecular ions directly from tissue in a spatially correlated manner [1]. Main factors contributing to the increasing popularity of this technology include the ability to (1) analyze a large range of molecular classes (proteins, peptides, lipids, metabolites, pharmaceuticals); (2) reveal disease-related biomolecular changes in highly localized regions; and (3) unravel changes that are invisible to established histopathological methods [2–4].

MSI has been applied to a multitude of tissues from various tumor tissues [5, 6] to plant tissues [7], but rodent brain [8, 9] is still the most frequently analyzed tissue type because of its widespread availability and use in neurologic research [10]. For instance, MALDI-MSI has been used to visualize spatiotemporal disturbances in rodent models of seizure [11], stroke [12], Alzheimer’s disease [13, 14], and Parkinson’s disease [15–17]. These studies demonstrate the potential of MSI for neurologic research, and which have been further validated by studies using small animal cohorts [8]. The application of MSI to preclinical investigations of neurologic disorders requires considerable multidisciplinary capabilities: MSI, statistics, and knowledge of brain anatomy. The latter aspect is exacerbated by the high degree of variation in brain region size that exists between animals [18] and the variability introduced during tissue sectioning and mounting. We recently demonstrated how MSI data could be automatically aligned to the Allen Brain Atlas, which allows the analyst to ensure all tissue sections of animals are obtained from a similar region of the mouse brain and to extract the mass spectral signatures from identical brain regions [19]. Here, we demonstrate how this registration pipeline enables larger-scale preclinical investigations of neurologic disorders. This first demonstration of such a cohort studied and analyzed by MSI concerns migraine.

Migraine is a common, severe episodic brain disorder that is characterized by attacks of severe unilateral throbbing headache associated with nausea, vomiting, photo- and phonophobia [20, 21]. An aura, which consists of transient neurologic symptoms, including visual and sensory disturbances, can accompany a migraine attack in one-third of patients. The aura is caused by cortical spreading depression (CSD), a slow, self-propagating wave of neuronal and glial cell depolarization in the cerebral cortex of one hemisphere followed by neuronal depression [22–24]. CSD causes a temporary dramatic failure of brain homeostasis, efflux of neurotransmitters, and changes in metabolism. Several metabolites, such as labile phosphate compounds (ATP, ADP, AMP, their cyclic analogues, cGMP, and phosphocreatine) and glycolytic metabolites (lactate, pyruvate, glucose, and glycogen) have been associated with CSD [25].

In 2012, Jones et al. [26] reported a proof-of-concept MALDI-MSI investigation of the biomolecular changes in C57BL/6 J wild-type (WT) mouse brain following CSD. The study included 2D- and 3D-MSI datasets, including an exclusion matrix to highlight apparent CSD-related changes, but omitted any form of statistical analysis because of the small number of animals involved in the study. Accordingly, the results obtained provided limited information regarding the biological aspects and changes associated with CSD. Here we report, for the first time, the application of MSI to a large scale animal cohort of a neurologic disease, in particular CSD as the neurobiologic correlate of the migraine aura. To this end, we make use of a relevant mouse model of migraine (i.e., knock-in transgenic mice carrying the pathogenic human R192Q missense mutation in the *Cacna1a* gene that encodes the α_1_ subunit of voltage-gated neuronal Ca_V_2.1 Ca^2+^ channels [27, 28]). Ca_V_2.1 Ca^2+^ channels with a R912Q-mutated α_1_ subunit cause familial hemiplegic migraine type 1 (FHM1) [29], a monogenic subtype of migraine with aura characterized by a prominent transient hemiparesis during the aura [20]. FHM1 R192Q mice exhibit an increased propensity to CSD, most likely because of an enhanced glutamatergic neurotransmission [27, 28, 30, 31]. In addition, unlike in WT mice, CSD waves can reach subcortical areas in R192Q mice, which correlate with the clinical phenotype [32]. We hypothesized that CSD could induce the expression of different biomolecular profiles in the brains of R192Q mice compared with wild-type. We measured and compared the biomolecular profiles of both mouse strains at specific cortical and subcortical brain regions and were able to show different consequences of CSD on the brains of R192Q and wild-type mice.

## Materials and Methods

### Animals

Male 2- to 4-month-old transgenic FHM1 R192Q mice (carrying the human pathogenic missense mutation R192Q) and corresponding non-transgenic wild-type (WT) mice were used. Transgenic mice were generated by introducing the human pathogenic mutation in the mouse *Cacna1a* gene using a gene targeting approach, as described in [27]. All mice were kept in a normal 12:12 light/dark regime and food and water were available ad libitum. The 32 animals used in this study were divided into different groups according to the experimental conditions: WT-Naïve (five animals); WT-Sham (six animals); WT-CSD (five animals); R192Q-Naïve (five animals); R192Q-Sham (six animals); R192Q-CSD (five animals). All experiments were approved by the Animal Experiment Ethics Committee of Leiden University Medical Center.

### CSD Experiments

CSD experiments were performed as previously described [26]. In brief, the mice were anesthetized with 4% isoflurane in pressurized air (21% O_2_ and 79% N_2_) and mounted on a stereotactic frame (David Kopf, Tujunga, CA, USA); 1.5% isoflurane was used for maintenance of the anesthesia. A midline incision was made to expose the skull. Two burr holes were drilled over the following coordinates (from bregma): 0.5 mm anterior, 2 mm lateral for DC recordings, and 3.5 mm posterior, 2 mm lateral for KCl or NaCl application. Seven CSDs were induced by applying a cotton ball soaked in 1 M KCl (CSD) or NaCl (Sham) for 30 s followed by extensive saline washing. The interval between two successive applications was 5 min. DC-potential signals were measured with respect to an Ag/AgCl reference electrode placed subcutaneously in the neck and amplified 10× (Molecular Devices, Sunnyvale, CA, USA). The DC signal was low-pass filtered at 4 Hz and digitized at 100–200 Hz using PowerLab 16/30 hardware (AD Instruments, Inc., Colorado Springs, CO, USA). Data were recorded and analyzed off-line using LabChart Pro (AD Instruments).

### Sample Collection and Tissue Preparation

Following seven CSD/Sham events, the mice were decapitated 5 min after the last CSD/Sham event, the brains quickly removed (within <2 min), immediately snap-frozen on powdered dry ice, and stored at –80°C until further processing. Coronal tissue sections, 12-μm thick, were cut at –12°C using a cryostat microtome (Leica Microsystems, Wetzlar, Germany), thaw-mounted onto poly-L-lysine coated indium-tin-oxide (ITO) glass slides (Bruker Daltonics, Bremen, Germany), and stored at –80°C. In order to exclude the effect of electrode insertion and KCl or NaCl application, only sections from the middle part of the brain (posterior from bregma, in between locations –1.22 and –1.94 mm) were used for MSI analysis. For the selection of sections in this part of the brain, the brain was trimmed in 25 μm slices and individual sections were visually inspected under a microscope and compared with the Paxinos Mouse Brain Atlas reference (3rd Edition; ISBN 978-0-12-374244-5) to judge their location along the anteroposterior axis, based on histologic landmarks. Once the level corresponding to the location –1.22 posterior from bregma was reached, 12 μm thick coronal sections were collected for the MSI experiments. For each animal, consecutive tissue sections were collected on different ITO slides for the analysis of proteins, peptides, and metabolites. Each ITO slide contained tissue sections from four animals. A semi-supervised block randomization was used to distribute the sections in a random way across and within slides while maximizing the group heterogeneity within a slide. This included the position of each sample on the MALDI slides and the measurement order within a slide—in order to minimize any potential sources of bias during MSI data acquisition (see Supplementary Information for the pseudo-code).

### Mass Spectrometry Imaging

Tissue sections were collected from storage at –80°C and equilibrated to room temperature (RT, 23°C) for 30 min in a vacuum desiccator. The slides were prepared for MALDI-MSI according to the molecular class to be analyzed. For peptide and protein imaging the tissue sections were washed as follows: (1) dip in 70% ethanol for 30 s; (2) dip in 96% ethanol for 30 s; (3) five short dips in deionized water; (4) dip in 70% ethanol for 30 s; (5) dip in 96% ethanol for 30 s; and finally (6) dried in a vacuum desiccator for 15 min. No washing procedure was applied to the samples used for the analysis of metabolites. MALDI matrix was uniformly applied over the brain sections using the SunCollect sprayer (SunChrom, Friedrichsdorf, Germany) according to the analyzed molecular class: sinapinic acid (SA; 5 mg/mL in 50% acetonitrile/0.3% TFA) was used for proteins; α-cyano-4-hydroxycinnamic acid (CHCA; 5 mg/mL in 50% acetonitrile/ 0.3% TFA) was used for peptides; and 9-aminoacridine (9AA; saturated solution in 70% methanol) was used for metabolites. MSI analyses of peptides (600–2000 Da) and metabolites (50–1000 Da) were performed using an UltrafleXtreme MALDI-TOF/TOF (Bruker Daltonics) in the reflectron positive (for peptides) or negative (for metabolites) ion mode with 100 μm raster width, 500 laser shots per pixel. MSI of proteins (3000–20,000 Da) was performed in an Autoflex III MALDI-TOF (Bruker Daltonics) in the linear positive ion mode with 100 μm raster width, 500 laser shots per pixel. Data acquisition, preprocessing, and visualization were performed using the flex software package from Bruker Daltonics: flexImaging 3.0 was used for experiment definition; flexControl 3.4 was used for data acquisition; and flexAnalysis 3.4 was used for on-the-fly mass spectral processing—metabolite/peptide datasets were preprocessed using a Gauss smoothing algorithm (width 0.02 *m/z*, 2 cycles) and a TopHat baseline subtraction algorithm; protein MSI spectra were preprocessed identically except the parameters of the Gauss smoothing algorithm were adapted for the lower mass resolution (width 2 *m/z*, 4 cycles).

After the MSI experiments, the matrix was washed off with 70% ethanol and the tissue samples stained with cresyl violet (Nissl staining). High-resolution histologic images were recorded using a Pannoramic MIDI digital slide scanner (3D Histech, Budapest, Hungary).

### Processing and Reduction of MSI Datasets

High-resolution histologic images were co-registered to the MSI data with flexImaging using fiducial markers applied at defined positions on each ITO slide with water-based correction fluid (Tipp-Ex; Ecolutions, BIC, Clichy, France) before MSI analyses. A list of all mass spectra contained within each brain section was extracted into an XML file for further processing in MATLAB R2011a (MathWorks, Natick, MA, USA). The preprocessed mass spectra contained in the MSI datasets were then read into Matlab.

#### Metabolite Datasets

The spectra were normalized to their total-ion-count (TIC) on a pixel-by-pixel basis and aligned on common peaks that are present in at least 85% of the samples. Peak picking and feature extraction was performed using the global base peak mass spectrum [33]. Briefly, this routine distils the original MSI data into an image cube containing the spatial distribution of every detected peak. Finally, a logarithmic-based variance-stabilizing transformation was applied to the peak intensities in order to reduce the impact of Poisson noise in the datasets [34, 35].

#### Peptide and Protein Datasets

Except for the logarithmic transformation of peak intensities, which was not performed for these datasets, all processing steps were as described above including TIC normalization on a pixel-by-pixel basis, with minor modifications in the thresholds used for peak picking to account for the different characteristics of the peptide and protein datasets.

### Image Processing and Registration to the Mouse Allen Brain Atlas

The reduced MSI datasets and the aligned histologic images were registered to the mouse Allen Brain Atlas (ABA); http://www.brain-map.org/) using our recently developed pipeline [19]. In brief, the histologic images are first preprocessed to reduce background noise and acquisition artifacts. Then the ABA corresponding histologic image is selected based on the maximum cord length of the hippocampus. Image registration is performed by applying a rigid affine transformation (correct for translation, shearing, rotation, and scaling), followed by nonlinear registration based on a B-Spline transform (correct for local deformations). Finally, the transformation matrix used to register the sample and ABA histologic images is applied to the respective MSI datasets.

### Anatomy Driven Data Analysis

The anatomic annotations contained in the ABA were used to define four anatomical regions of interest (ROI) in the MSI datasets: cortex (C), striatum (S), hippocampus (H), and thalamus (T). MS data were extracted from each ROI from every ABA-aligned MSI dataset for statistical analysis: (1) a non-paired Student’s *t*-test was used for comparisons between independent groups; and (2) a paired Student’s *t*-test was used for comparisons (left versus right hemispheres) within each independent group of animals. The Benjamini-Hochberg procedure was used to correct for multiple testing. All statistical analyses were done in R (R Foundation for Statistical Computing, Vienna, Austria) and Matlab, in which *P*-values < 0.05 were considered statistically significant.

## Results

### CSD Induction and MSI Analysis

Seven CSD events were evoked, each with a 5 min interval in the occipital cortex of WT and transgenic FHM1 R192Q mice; equivalent Sham experiments utilizing aqueous NaCl instead of aqueous KCl, which does not evoke a CSD, were performed in parallel so as to clearly differentiate CSD-related from non-CSD-related biomolecular changes. As shown in Table S1 (Supplementary Information), there were no significant differences between R192Q and WT mice regarding the CSD characteristics and time under anesthesia. All animals were sacrificed 5 min after the last CSD event and the brains were immediately removed and frozen on dry ice to limit post-mortem degradation effects. Previous studies have indicated that the analysis of metabolites can be significantly impaired by post-mortem degradation [36–38]. These results indicated that labile metabolites, such as AMP, ADP, and ATP that act as energy reserves in the brain can be used as a measure of the post-mortem degradation effects. We measured the AMP/ATP ratios across the different mouse brains but found no correlation with the short post-mortem times of these experiments, which indicated that any variability associated with the isolation of the mouse brain had a negligible effect on the MSI data.

### Alignment of MSI Datasets to a Single Reference System

**Figure 1:**
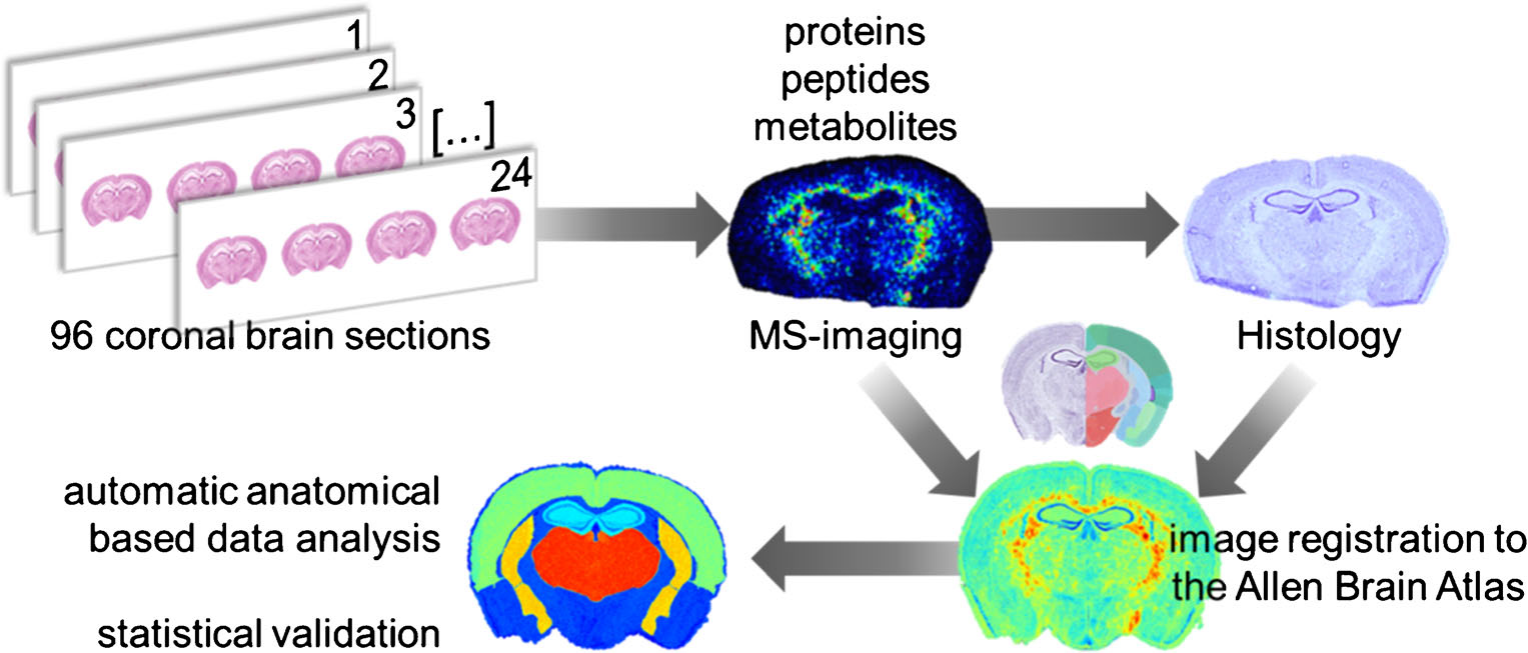
Schematic of workflow developed to analyze the effect of CSD in WT and R192Q mouse brains. Ninety-six coronal brain sections were obtained from a total of 32 mouse brains (three consecutive sections per animal). Proteins, peptides, and metabolites were independently analyzed by MSI using optimized sample treatment for each molecular class as described in the Materials and Methods section. Each section was stained with Nissl reagent after matrix removal and the MSI datasets, and histologic images were aligned to the Allen Brain Atlas of mouse brain [19]. Automatic anatomical annotation of regions of interest allowed the extraction of MSI data from specific brain regions of interest and statistical analysis

Preclinical studies typically compare a number of animals per test group to guard against the individual variation in any animal population. We have analyzed a total of 96 mouse brain sections from 32 animals from 16 R192Q and 16 WT mice. We registered all histologic images and MSI datasets to the mouse brain reference atlas contained within the Allen Brain Atlas [19] in order to (1) reduce the impact of variance attributable to differences in brain region size [18]; (2) check if all tissue sections came from a similar region of the brain; and (3) correct for any tissue-processing artifacts introduced during the experiment (e.g. ,folds, tears). The 96 brain sections were registered to just 3 of 132 different coronal sections present in the ABA reference atlas, corresponding to a tissue-section sampling accuracy between animals of 200 μm, and thus indicating we sampled similar regions of the mouse brain. The registration itself was performed with an accuracy of less than 30 μm, which is below the spatial resolution used for the MSI measurements (100 μm) (see Figure 5, reference [19]). A scheme of the workflow is presented in Figure 1.

### Anatomy Based Data Analysis

The distribution of proteins, peptides, and metabolites after CSD and Sham operations was investigated across the whole brain and in four particular brain regions that are of relevance to migraine pathophysiology [32]: cortex, striatum, hippocampus, and thalamus. Electrophysiology measurements indicate that when induced in one hemisphere, CSD does not cross to the other hemisphere [30, 39]; accordingly, the left (unaffected) hemisphere was used as control for the CSD/Sham-affected right hemisphere.

#### Protein Datasets

**Figure 2:**
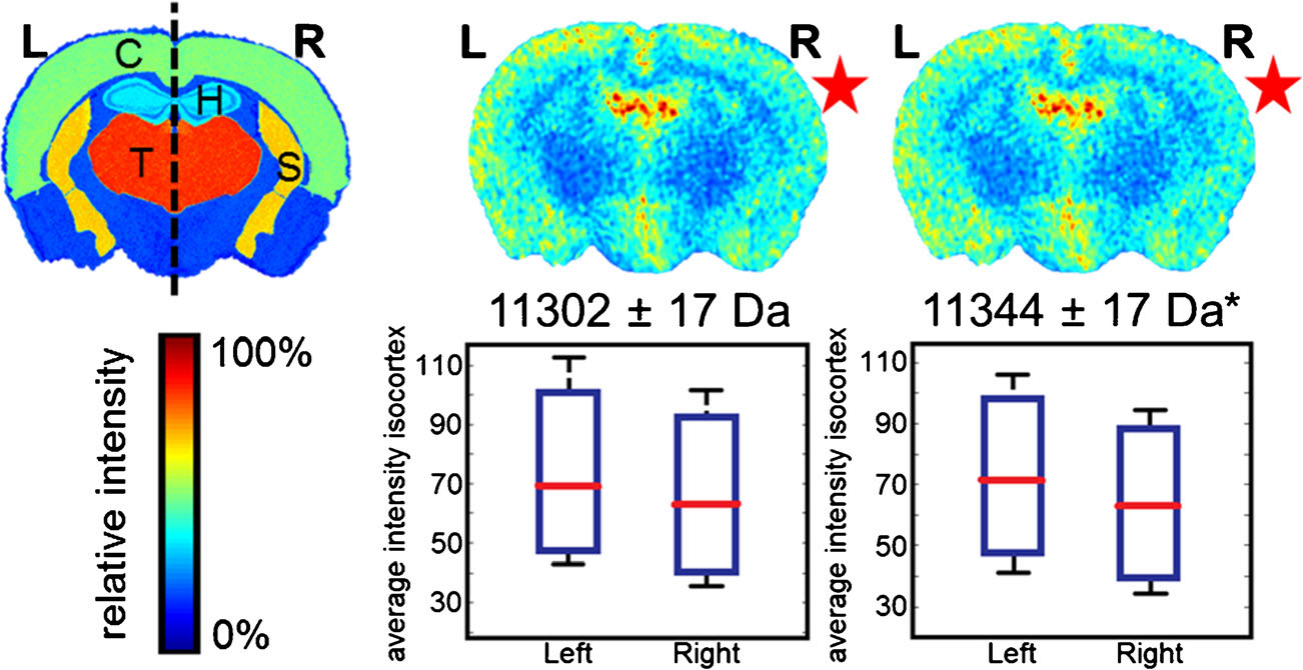
Protein MSI dataset: differences between CSD (right, R) and control hemisphere (left, L) in R192Q-CSD mouse brain. Each image corresponds to the visualization of the average distribution of a particular *m/z* feature in five mouse brains after alignment to the ABA (*P < 0.05, Student’s *t*-test). C = cortex; T = thalamus; H = hippocampus; S = Striatum

Comparison between right (CSD-affected) and left (control) hemispheres within the R192Q-CSD group revealed moderate differences in the distribution of *m/z* feature 11,302 Da and statistically significant differences in the distribution of *m/z* feature 11,344 Da (*P* < 0.05, Student’s *t*-test), as shown in Figure 2. In both cases, the ion intensities are lower in the CSD-affected hemisphere (R). These changes were not observed in the WT and Sham groups. Poté et al. [40] have previously identified the same molecular features as histone H4 and respective acetylated form while analyzing hepatocellular carcinoma. Although their experiment concerned human tissue samples, a Basic Local Alignment Search Tool (BLAST) search (in UniProt database) revealed 100% homology with murine histone H4.

#### Peptide Datasets

**Figure 3:**
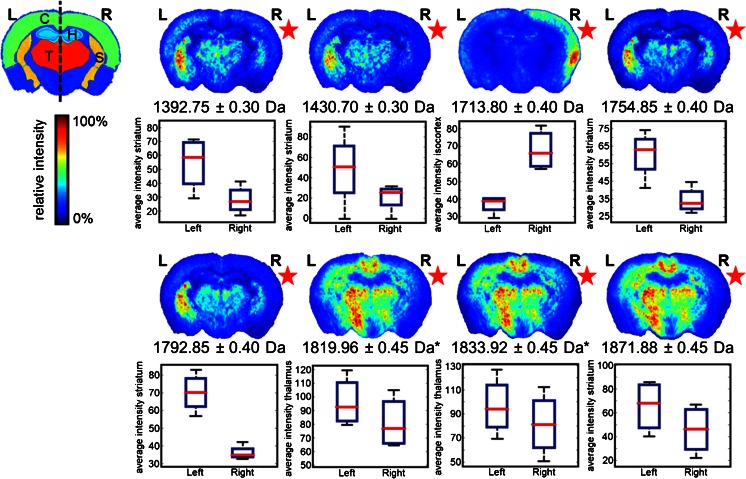
Peptide MSI dataset: differences between CSD (right, R) and control hemisphere (left, L) in R192Q-CSD mouse brain. Each image corresponds to the visualization of the average distribution of a particular *m/z* feature in five mouse brains after alignment to the ABA (*P < 0.05, Student’s *t*-test). C = cortex; T = thalamus; H = hippocampus; S = striatum

Intra-group comparison between right (CSD-affected) and left (control) hemispheres showed significant differences occurring in the thalamus region for *m/z* features 1819.96 Da and 1833.96 Da (*P* < 0.05, Student’s *t*-test) only in R192Q-CSD mice (Figure 3). A decrease in ion intensities was observed in the CSD-affected brain hemisphere in both cases. Similarly to the protein dataset, also these biomolecular features share the same spatial distribution and are separated by 14 Da, which is compatible with a methylation post-translational modification. Moderate differences were also observed in the R192Q mouse brains after CSD, in particular *m/z* 1713.8 with increased expression in the cortex of the CSD-affected hemisphere, and *m/z* 1754.85 and its K^+^ adduct *m/z* 1792.85 with decreased expression in the striatum of the CSD hemisphere. No significant biomolecular changes were observed for the Sham and Naïve mouse groups.

#### Metabolite Datasets

**Figure 4:**
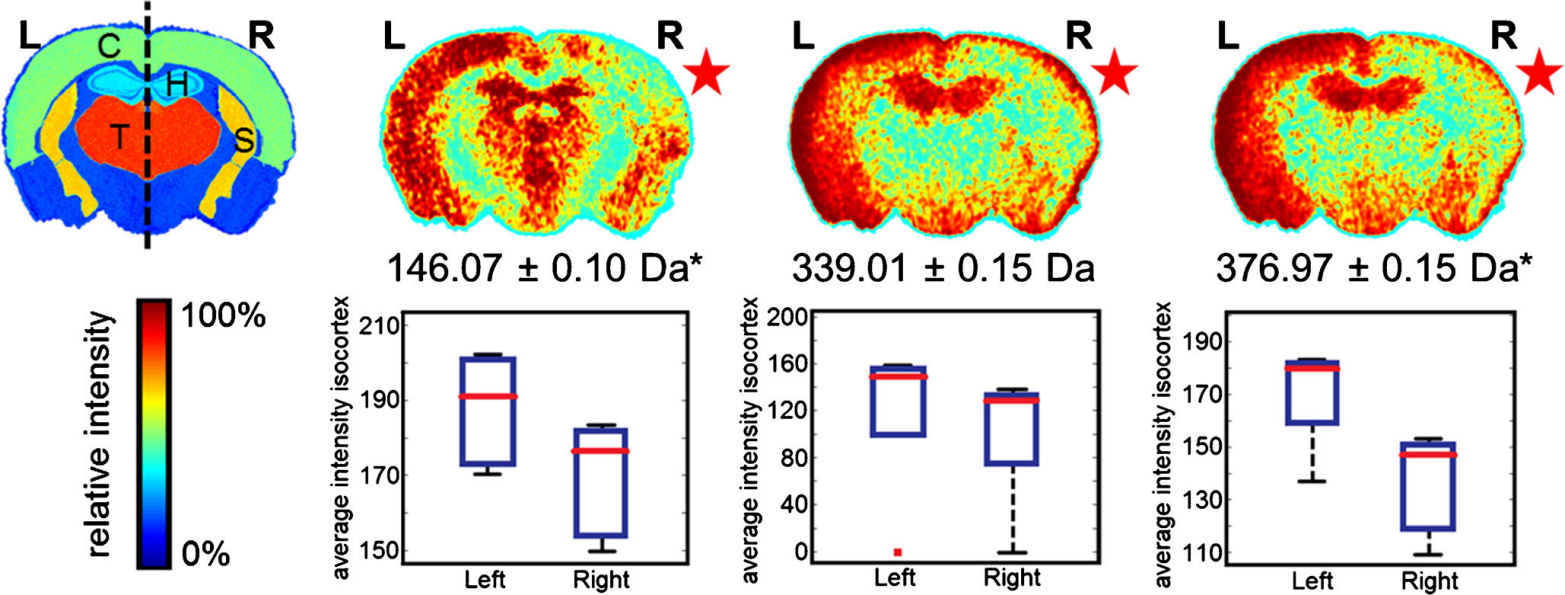
Metabolite MSI dataset: differences between CSD (right, R) and control hemisphere (left, L) in R192Q-CSD mouse brain. Each image corresponds to the visualization of the average distribution of a particular *m/z* feature in five mouse brains after alignment to the ABA (*P < 0.05, Student’s *t*-test). C = cortex; T = thalamus; H = hippocampus; S = striatum

Intra-group comparisons between control and CSD-affected hemispheres revealed differences associated with CSD in WT and R192Q mice (Supplementary Information, Figure S1). Biomolecular features present at 89.03 Da showed an increase in signal intensity in the CSD-affected hemisphere, whereas *m/z* features at 146.07 Da, 339.01 Da, 360.97 Da, and 376.97 Da presented a decrease in intensity in the CSD-affected hemisphere. Interestingly, significant changes were only found in the distribution of two *m/z* features for R192Q-CSD mice (*P* < 0.05, Student’s *t*-test), particularly *m/z* 146.07 Da and 376.97 Da as shown in Figure 4. In order to more confidently assign the *m/z* features observed, we performed high-resolution MS analysis by MALDI Fourier transform ion cyclotron resonance (FTICR) directly from tissue. Accurate masses were then searched in the metabolite database METLIN revealing the presence of glutamate ([M – H]^–^ 146.0459 Da), fructose 1,6-bisphosphate ([M – H]^–^ 338.9888 Da), and fructose 1,6-bisphosphate K^+^ adduct ([M + K – 2H]^–^ 376.9447 Da) among other isobaric species.

## Discussion

MSI is a non-targeted methodology that allows the analysis of different biomolecular classes directly from tissue. One of the main advantages of MSI is its ability to unravel biomolecular changes independently of histology. This is of particular interest to the study of migraine, a neurologic disorder characterized by recurrent attacks and lack of clear histopathologic features. Many metabolites, amino acids, and neuropeptides have been measured in the brain during and after CSD experiments [25]. However, the effects of CSD are still not fully understood, namely: (1) How are the proteome, peptidome, and metabolome profiles affected by CSD? (2) How are the CSD changes affected by the genetic background of the mice (i.e., a comparison between mice with a genetic predisposition to migraine and wild-type mice)? (3) Does CSD induce biomolecular changes in subcortical areas? To answer these questions, we measured and compared the biomolecular profiles of FHM1 R192Q and WT mice after CSD induction in the occipital cortex.

The biomolecular profiles recorded by MSI presented disturbances that may be associated with the CSD wave progression only in R192Q mouse brain. Given the short time between CSD induction and animal sacrifice, ca. 40 min after the first CSD event, significant changes in protein expression level were not expected. Indeed, the only significant change revealed by MSI is associated with a post-translational modification in histone H4: acetylated histone H4 (11,344 Da) showed a decreased intensity in the cortical region of the CSD-affected hemisphere. Likewise, previous reports showed that CSD induction in rats affected methylation levels in the cortex, although this was evident 24 h following CSD induction [41, 42]. Histone modifications such as acetylation, methylation, ubiquitination, and phosphorylation have an important role in the epigenetic regulation of transcription and have been associated with neurologic diseases such as Alzheimer's disease, Huntington’s disease, and Parkinson’s disease [43]. Therefore, the effect of CSD on post-translational modification of histones and, consequently, on the transcription mechanisms, may have relevance to migraine.

It is well known that CSD triggers the release of vasoactive peptides [44, 45]. Trigeminal axons that innervate the dural vessels and are activated during CSD release calcitonin gene related peptide (CGRP), Substance P (SP), and neurokinin-α (NKA), which all are potent vasodilators [45–49]. These vasoactive peptides are believed to be mediators of neurogenic inflammation, which is thought to be a mechanism relevant to the generation of migraine headache [48, 49]. Interestingly, levels of CGRP and SP in the blood of migraine patients were found increased between and during attacks [50–52]. Although we did not observe significant changes in any of these compounds, the MSI peptide datasets revealed moderate differences in the distributions of a few biomolecular features (1392.75 Da; 1713.80 Da; 1754.85 Da; 1871.88 Da) in mouse brain hemispheres affected by CSD, which is in agreement with the idea that CSD induction triggers a substantial redistribution of peptides in the extracellular space. Additionally, a significant decrease in the intensity of *m/z* features 1819.96 and 1833.92 Da was observed only in the thalamus region of R192Q mice. Although the identity of these *m/z* features is currently unknown, their similar distribution and *m/z* shift consistent with a methylation post-translational modification indicate that these may be different forms of the same peptide.

In the peptide datasets, we detected changes in subcortical regions (striatum and thalamus) as a result of CSD. This is in agreement with previously published data indicating that CSD induction in the cortex may also easily spread to subcortical regions in FHM1 R192Q but not in wild-type mice [32]. Therefore, the differential distribution of peptides in the striatum, thalamus, and hippocampus identified after CSD might reflect a subcortical spread of CSD waves. Yet, the possibility for a transport of peptides released in the cortex to subcortical structures cannot be excluded at this time.

Several metabolites such as ATP, ADP, AMP, cGMP, lactate, pyruvate, glucose, and glycogen have been associated with CSD [25]. In addition, CSD can also trigger the release of amino acids and change their brain regional distribution. During single episodes of CSD in rat brain, interstitial levels of several amino acids (e.g., alanine, arginine, aspartate, glutamate, glycine) were found to be elevated, highlighting the massive changes in biomolecular distribution that occur in the brain during episodes of CSD [53]. Our MSI metabolite datasets revealed a significant decrease in the intensity of *m/z* feature 146.07 in the cortical region of R192Q mice after CSD. Owing to the high number of isobaric molecules in this mass region, it is virtually impossible to identify small metabolites directly from tissue with MS/MS analysis. Yet, after high-resolution MS analysis and assignment based on previous reports, this biomolecule was assigned as glutamate. Glutamate plays a major role in CSD, and elevated levels of glutamate and glutamic acids have been detected in plasma [54] and cerebrospinal fluid (CSF) [55, 56] of migraine patients. Of note, pharmacologic targeting of glutamate receptors is currently being explored as a potential migraine therapy [57]. Glutamate is a well-known trigger of CSD [58], and during CSD propagation in the cortex there is a release of glutamate to the extracellular space [53, 59]. Given the above, the down-regulation of glutamate in cortex of CSD-affected brain hemispheres observed by MSI analysis may seem contradictory, at first. Previous quantitative proteomics studies in naïve FHM1 R192Q mice, however, revealed an up-regulation of major glutamate transporters, EAAT1 and EAAT2, compared with WT mice [60]. These findings, together with the MSI results reported here, suggest that a compensatory mechanism in the brain might be in place to clear excessive glutamate from the synaptic space by glial cells using glutamate transporters.

Besides the putative identification of glutamate changes, our MSI data revealed significant differences in the spatial localization of *m/z* 376.96 Da in the R192Q brain after CSD. Database search based on high-resolution MS indicated that this molecular feature is probably associated with different forms of fructose 1,6-biphosphate, which is a byproduct of fructose and glucose metabolism common to all cells. In a previous publication, this *m/z* feature was also assigned as fructose 1,6-bisphospate by MSI in a rat model of ischemic stroke [12]. Interestingly, the distribution of fructose 1,6-biphosphate and glutamate in the ischemic brain is similar to the distribution observed after CSD in both R192Q and WT mice. Recently Eikermann-Haerter et al. [61] studied the link between stroke and migraine using the same transgenic FHM1 R192Q mice and demonstrated that FHM mutations do not only enhance susceptibility to CSD but also to ischemic depolarizations, leading to stroke. Therefore, the MSI results reported here in combination with previous work by Miura et al. [12] seem to suggest that there is indeed a link between CSD and stroke events, although more work is required to prove this hypothesis.

## Conclusions

Here we used MALDI-MSI combined with a newly developed pipeline that allows the automatic registration of MS datasets to mouse data contained in the Allen Brain Atlas [19], to investigate the biomolecular distribution in the brain after CSD in a relevant mouse model of migraine. Our results revealed that CSD events affect the distribution of metabolites, peptides and proteins, not only in the cortex but also in subcortical structures. The finding that changes in biomolecules distribution were only evident in R192Q mice that had undergone CSD indicates that these changes are both genotype- and CSD-specific. Future work should reveal the identities of biomolecules that are affected by CSD events and might provide more in-depth insights in migraine pathophysiology. In conclusion, our results show that CSD induction in FHM1 R192Q mice is associated with a substantial redistribution of biomolecules in the brain and highlight that MALDI-MSI can be instrumental in preclinical animal models of disease.

## Electronic supplementary material

ESM 1 (DOCX 1519 kb)

## References

[1] Caprioli RM, Farmer TB, Gile J (1997). Molecular imaging of biological samples: localization of peptides and proteins using MALDI-TOF MS. Anal. Chem..

[2] McDonnell LA, Heeren RM, Andrén PE, Stoeckli M, Corthals GL (2012). Going forward: increasing the accessibility of imaging mass spectrometry. J. Proteome..

[3] Norris JL, Caprioli RM (2013). Analysis of tissue specimens by matrix-assisted laser desorption/ionization imaging mass spectrometry in biological and clinical research. Chem. Rev..

[4] McDonnell LA, Heeren RMA (2007). Imaging mass spectrometry. Mass Spectrom. Rev..

[5] McDonnell LA, Corthals GL, Willems SM, van Remoortere A, van Zeijl RJ, Deelder AM (2010). Peptide and protein imaging mass spectrometry in cancer research. J. Proteome..

[6] Balluff B, Schöne C, Höfler H, Walch A (2011). MALDI imaging mass spectrometry for direct tissue analysis: technological advancements and recent applications. Histochem. Cell Biol..

[7] Kaspar S, Peukert M, Svatos A, Matros A, Mock H-PP (2011). MALDI-imaging mass spectrometry—an emerging technique in plant biology. Proteomics.

[8] Hanrieder J, Phan NTN, Kurczy ME, Ewing AG (2013). Imaging mass spectrometry in neuroscience. ACS Chem. Neurosci..

[9] Shariatgorji M, Svenningsson P, Andrén PE (2014). Mass spectrometry imaging, an emerging technology in neuropsychopharmacology. Neuropsychopharmacology.

[10] Hafezparast M, Ahmad-Annuar A, Wood NW, Tabrizi SJ, Fisher EM (2002). Mouse models for neurological disease. Lancet Neurol..

[11] Sugiura Y, Taguchi R, Setou M (2011). Visualization of spatiotemporal energy dynamics of hippocampal neurons by mass spectrometry during a kainate-induced seizure. PLoS One.

[12] Miura D, Fujimura Y, Yamato M, Hyodo F, Utsumi H, Tachibana H, Wariishi H (2010). Ultrahighly sensitive in situ metabolomic imaging for visualizing spatiotemporal metabolic behaviors. Anal. Chem..

[13] Stoeckli M, Knochenmuss R, McCombie G, Mueller D, Rohner T, Staab D, Wiederhold K-HH (2006). MALDI MS imaging of amyloid. Methods Enzymol..

[14] Rohner TC, Staab D, Stoeckli M (2005). MALDI mass spectrometric imaging of biological tissue sections. Mech. Ageing Dev..

[15] Pierson J, Norris JL, Aerni HR, Svenningsson P, Caprioli RM, Andrén PE (2004). Molecular profiling of experimental parkinson’s disease: direct analysis of peptides and proteins on brain tissue sections by maldi Mass Spectrometry. J. Proteome Res..

[16] Nilsson A, Sköld K, Sjögren B, Svensson M, Pierson J, Zhang X, Caprioli RM, Buijs J, Persson B, Svenningsson P, Andrén PE (2007). Increased striatal mRNA and protein levels of the immunophilin FKBP-12 in experimental Parkinson’s disease and identification of FKBP-12-binding proteins. J. Proteome Res..

[17] Ljungdahl, A., Hanrieder, J., Fälth, M., Bergquist, J., Andersson, M.: Imaging mass spectrometry reveals elevated nigral levels of dynorphin neuropeptides in L-DOPA-induced dyskinesia in rat model of Parkinson’s disease. PloS one **6**, e25653 (2011) 10.1371/journal.pone.0025653 PMC3184165 21984936

[18] Hager, R., Lu, L., Rosen, G.D., Williams, R.W.: Genetic architecture supports mosaic brain evolution and independent brain-body size regulation. Nat. Commun. **3**, 1079 (2012) 10.1038/ncomms2086 PMC4267555 23011133

[19] Abdelmoula WM, Carreira RJ, Shyti R, Balluff B, van Zeijl RJ, Tolner EA, Lelieveldt BF, van den Maagdenberg AM, McDonnell LA, Dijkstra J (2014). Automatic registration of mass spectrometry imaging data sets to the Allen brain atlas. Anal. Chem..

[20] ICHD (2004). The International Classification of Headache Disorders: 2nd edition. Cephalalgia.

[21] Goadsby PJ, Lipton RB, Ferrari MD (2002). Migraine-current understanding and treatment. N. Engl. J. Med..

[22] Hadjikhani N, Sanchez Del Rio M, Wu O, Schwartz D, Bakker D, Fischl B, Kwong KK, Cutrer FM, Rosen BR, Tootell RB, Sorensen AG, Moskowitz MA (2001). Mechanisms of migraine aura revealed by functional MRI in human visual cortex. Proc. Natl. Acad. Sci. U. S. A..

[23] Lauritzen M (1994). Pathophysiology of the migraine aura. The spreading depression theory. Brain.

[24] Pietrobon D, Moskowitz MA (2013). Pathophysiology of migraine. Annu. Rev. Physiol..

[25] Selman WR, Lust WD, Pundik S, Zhou YN, Ratcheson RA (2004). Compromised metabolic recovery following spontaneous spreading depression in the penumbra. Brain Res..

[26] Jones EA, Shyti R, van Zeijl RJ, van Heiningen SH, Ferrari MD, Deelder AM, Tolner EA, van den Maagdenberg AM, McDonnell LA (2012). Imaging mass spectrometry to visualize biomolecule distributions in mouse brain tissue following hemispheric cortical spreading depression. J. Proteome..

[27] van den Maagdenberg AM, Pietrobon D, Pizzorusso T, Kaja S, Broos LA, Cesetti T, van de Ven RC, Tottene A, van der Kaa J, Plomp JJ, Frants RR, Ferrari MD (2004). A *Cacna1a* knock-in migraine mouse model with increased susceptibility to cortical spreading depression. Neuron.

[28] Ferrari MD, Klever RR, Terwindt GM, Ayata C, van den Maagdenberg AM (2015). Migraine pathophysiology: lessons from mouse models and human genetics. Lancet Neurol..

[29] Ophoff RA, Terwindt GM, Vergouwe MN, van Eijk R, Oefner PJ, Hoffman SM, Lamerdin JE, Mohrenweiser HW, Bulman DE, Ferrari M, Haan J, Lindhout D, van Ommen GJ, Hofker MH, Ferrari MD, Frants RR (1996). Familial hemiplegic migraine and episodic ataxia type-2 are caused by mutations in the Ca2+ channel gene CACNL1A4. Cell.

[30] Eikermann-Haerter K, Dilekoz E, Kudo C, Savitz SI, Waeber C, Baum MJ, Ferrari MD, van den Maagdenberg AM, Moskowitz MA, Ayata C (2009). Genetic and hormonal factors modulate spreading depression and transient hemiparesis in mouse models of familial hemiplegic migraine type 1. J. Clin. Invest..

[31] Tottene A, Conti R, Fabbro A, Vecchia D, Shapovalova M, Santello M, van den Maagdenberg AM, Ferrari MD, Pietrobon D (2009). Enhanced excitatory transmission at cortical synapses as the basis for facilitated spreading depression in Ca(v)2.1 knock-in migraine mice. Neuron.

[32] Eikermann-Haerter K, Yuzawa I, Qin T, Wang Y, Baek K, Kim YR, Hoffmann U, Dilekoz E, Waeber C, Ferrari MD, van den Maagdenberg AMJM, Moskowitz MA, Ayata C (2011). Enhanced subcortical spreading depression in familial hemiplegic migraine type 1 mutant mice. J. Neurosci..

[33] McDonnell LA, van Remoortere A, de Velde N, van Zeijl RJ, Deelder AM (2010). Imaging mass spectrometry data reduction: automated feature identification and extraction. J. Am. Soc. Mass Spectrom..

[34] Veselkov KA, Mirnezami R, Strittmatter N, Goldin RD, Kinross J, Speller AV, Abramov T, Jones EA, Darzi A, Holmes E, Nicholson JK, Takats Z (2014). Chemo-informatic strategy for imaging mass spectrometry-based hyperspectral profiling of lipid signatures in colorectal cancer. Proc. Natl. Acad. Sci. U. S. A..

[35] Keenan MR, Kotula PG (2004). Accounting for Poisson noise in the multivariate analysis of ToF-SIMS spectrum images. Surf. Interface Anal..

[36] Hattori K, Kajimura M, Hishiki T, Nakanishi T, Kubo A, Nagahata Y, Ohmura M, Yachie-Kinoshita A, Matsuura T, Morikawa T, Nakamura T, Setou M, Suematsu M (2010). Paradoxical ATP elevation in ischemic penumbra revealed by quantitative imaging mass spectrometry. Antioxid. Redox Signal..

[37] Sugiura Y, Honda K, Kajimura M, Suematsu M (2014). Visualization and quantification of cerebral metabolic fluxes of glucose in awake mice. Proteomics.

[38] Blatherwick EQ, Svensson CI, Frenguelli BG, Scrivens JH (2013). Localization of adenine nucleotides in heat-stabilised mouse brains using ion mobility enabled MALDI imaging. Int. J. Mass Spectrom..

[39] Somjen GG (2001). Mechanisms of spreading depression and hypoxic spreading depression-like depolarization. Physiol. Rev..

[40] Poté N, Alexandrov T, Le Faouder J, Laouirem S, Léger T, Mebarki M, Belghiti J, Camadro J-MM, Bedossa P, Paradis V (2013). Imaging mass spectrometry reveals modified forms of histone H4 as new biomarkers of microvascular invasion in hepatocellular carcinomas. Hepatology.

[41] Passaro D, Rana G, Piscopo M, Viggiano E, De Luca B, Fucci L (2010). Epigenetic chromatin modifications in the cortical spreading depression. Brain Res..

[42] Rana G, Donizetti A, Virelli G, Piscopo M, Viggiano E, De Luca B, Fucci L (2012). Cortical spreading depression differentially affects lysine methylation of H3 histone at neuroprotective genes and retrotransposon sequences. Brain Res..

[43] Konsoula, Z., Barile, F. A.: Epigenetic histone acetylation and deacetylation mechanisms in experimental models of neurodegenerative disorders. J. Pharmacol. Toxicol. Methods **66**, 215–220 (2012) 10.1016/j.vascn.2012.08.001 22902970

[44] Tozzi A, de Iure A, Di Filippo M, Costa C, Caproni S, Pisani A, Bonsi P, Picconi B, Cupini LM, Materazzi S, Geppetti P, Sarchielli P, Calabresi P (2012). Critical role of calcitonin gene-related peptide receptors in cortical spreading depression. Proc. Natl. Acad. Sci. U. S. A..

[45] Colonna DM, Meng W, Deal DD, Busija DW (1994). Calcitonin gene-related peptide promotes cerebrovascular dilation during cortical spreading depression in rabbits. Am. J. Physiol..

[46] Ho TW, Edvinsson L, Goadsby PJ (2010). CGRP and its receptors provide new insights into migraine pathophysiology. Nat. Rev. Neurol..

[47] Raddant AC, Russo AF (2011). Calcitonin gene-related peptide in migraine: intersection of peripheral inflammation and central modulation. Expert Rev. Mol. Med..

[48] Wahl M, Schilling L, Parsons AA, Kaumann A (1994). Involvement of calcitonin gene-related peptide (CGRP) and nitric oxide (NO) in the pial artery dilatation elicited by cortical spreading depression. Brain Res..

[49] Bolay H, Reuter U, Dunn AK, Huang Z, Boas DA, Moskowitz MA (2002). Intrinsic brain activity triggers trigeminal meningeal afferents in a migraine model. Nat. Med..

[50] Fusayasu E, Kowa H, Takeshima T, Nakaso K, Nakashima K (2007). Increased plasma substance P and CGRP levels, and high ACE activity in migraineurs during headache-free periods. Pain.

[51] Goadsby PJ, Edvinsson L, Ekman R (1990). Vasoactive peptide release in the extracerebral circulation of humans during migraine headache. Ann. Neurol..

[52] Gallai V, Sarchielli P, Floridi A, Franceschini M, Codini M, Glioti G, Trequattrini A, Palumbo R (1995). Vasoactive peptide levels in the plasma of young migraine patients with and without aura assessed both interictally and ictally. Cephalalgia.

[53] Fabricius M, Jensen LH, Lauritzen M (1993). Microdialysis of interstitial amino acids during spreading depression and anoxic depolarization in rat neocortex. Brain Res..

[54] Ferrari MD, Odink J, Bos KD, Malessy MJ, Bruyn GW (1990). Neuroexcitatory plasma amino acids are elevated in migraine. Neurology.

[55] Martinez F, Castillo J, Rodriguez JR, Leira R, Noya M (1993). Neuroexcitatory amino acid levels in plasma and cerebrospinal fluid during migraine attacks. Cephalalgia.

[56] Peres MF, Zukerman E, Senne Soares CA, Alonso EO, Santos BF, Faulhaber MH (2004). Cerebrospinal fluid glutamate levels in chronic migraine. Cephalalgia.

[57] Andreou AP, Goadsby PJ (2009). Therapeutic potential of novel glutamate receptor antagonists in migraine. Expert Opin. Investig. Drugs.

[58] Van Harreveld A (1959). Compounds in brain extracts causing spreading depression of cerebral cortical activity and contraction of crustacean muscle. J. Neurochem..

[59] Basarsky TA, Feighan D, MacVicar BA (1999). Glutamate release through volume-activated channels during spreading depression. J. Neurosci..

[60] Klychnikov OI, Li KW, Sidorov IA, Loos M, Spijker S, Broos LA, Frants RR, Ferrari MD, Mayboroda OA, Deelder AM, Smit AB, van den Maagdenberg AM (2010). Quantitative cortical synapse proteomics of a transgenic migraine mouse model with mutated Ca(V)2.1 calcium channels. Proteomics.

[61] Eikermann-Haerter K, Lee JH, Yuzawa I, Liu CH, Zhou Z, Shin HK, Zheng Y, Qin T, Kurth T, Waeber C, Ferrari MD, van den Maagdenberg AM, Moskowitz MA, Ayata C (2012). Migraine mutations increase stroke vulnerability by facilitating ischemic depolarizations. Circulation.

