# Supplementary material for: Large-Scale Mass Spectrometry Imaging Investigation of Consequences of Cortical Spreading Depression in a Transgenic Mouse Model of Migraine
Source: J Am Soc Mass Spectrom. 2015 Apr 16;26(6):853–61. doi: 10.1007/s13361-015-1136-8 (PMC4422864; doi:10.1007/s13361-015-1136-8)

Supplementary information:

Large-Scale Mass Spectrometry Imaging Investigation of Consequences of Cortical Spreading Depression in a Transgenic Mouse Model of Migraine

Ricardo J. Carreira^1,*^, Reinald Shyti^2,*^, Benjamin Balluff^1^, Walid M. Abdelmoula^3^, Sandra H. van Heiningen^2^, Rene J. van Zeijl^1^, Jouke Dijkstra^3^, Michel D. Ferrari^4^, Else A. Tolner^4^, Liam A. McDonnell^1,5**^, Arn M.J.M. van den Maagdenberg^2,4**^

* and ** equal contributions

^1^ Center for Proteomics and Metabolomics, Leiden University Medical Center, Leiden, Netherlands

^2^ Department of Human Genetics, Leiden University Medical Center, Leiden, Netherlands

^3^ Division of Image Processing, Department of Radiology, Leiden University Medical Center, Leiden, Netherlands

^4^ Department of Neurology, Leiden University Medical Center, Leiden, Netherlands

^5^ Fondazione Pisana per la Scienza ONLUS, Pisa, Italy

Corresponding authors and reprint requests

Dr. Liam A. McDonnell, Center for Proteomics and Metabolomics, Leiden University Medical Center, Einthovenweg 20, 2333 ZC Leiden, The Netherlands; E-mail: L.A.Mcdonnell@lumc.nl; Phone: +31 71 526 8744; Fax: +31 71 526 6907

**Table S1.** CSD characteristics and average time under anesthesia for the different mouse groups. Seven CSD events were evoked by topical application of 1M KCl in the occipital cortex of the right hemisphere of the brain with 5 minutes interval (equivalent Sham experiments were performed with topical application of 1M NaCl). Significant differences between R192Q and WT mice regarding the CSD characteristics and time under anesthesia were not observed.

| Group | N | Amplitude  (mV) | Duration  (sec) | Time anesthesia  (min) |
| --- | --- | --- | --- | --- |
| WT-CSD | 5 | 22.9±2.4 | 44.2±19.4 | 62.0±6.3 |
| WT-Sham | 6 |  |  | 62.0±2.2 |
| R192Q-CSD | 5 | 22.0±1.2 | 31.2±3.3 | 61.5±2.9 |
| R192Q-Sham | 6 |  |  | 65.1±4.3 |

**Pseudo-code of the semi-supervised block randomization**

- ***s*** = number of samples
- Define ***n*** * ***m*** experimental design matrix ***M***

***m*** = maximum number of samples that can be placed on a slide

***n*** = number of slides needed for the project = ceiling(***s***/***m***)

- Sort groups into list ***L*** according to amount of samples
- Fill up ***M*** iteratively by column
  - As long as there are empty places in a column, do
    - select next group from ***L***
    - draw randomly ***x*** samples from the selected group where ***x*** <= ***n***
    - place the selected samples randomly within the empty positions in the column
    - delete already taken samples from the initial sample list
- When done, permute samples within slides

**Figure S1:** The effect of CSD on the distribution of a number of metabolites in coronal sections of WT and R192Q mouse brain. Seven CSDs (and equivalent Sham experiments) were evoked in the right (R) hemisphere of WT and R192Q mouse brain. Twelve μm thick coronal brain sections were homogeneously sprayed with 9AA matrix and analyzed by MALDI-TOF-MS in the negative mode. MS-images refer to a single mouse example per experimental group. Lightning bolt indicates the hemisphere (right hemisphere) where the CSD event was evoked.

**
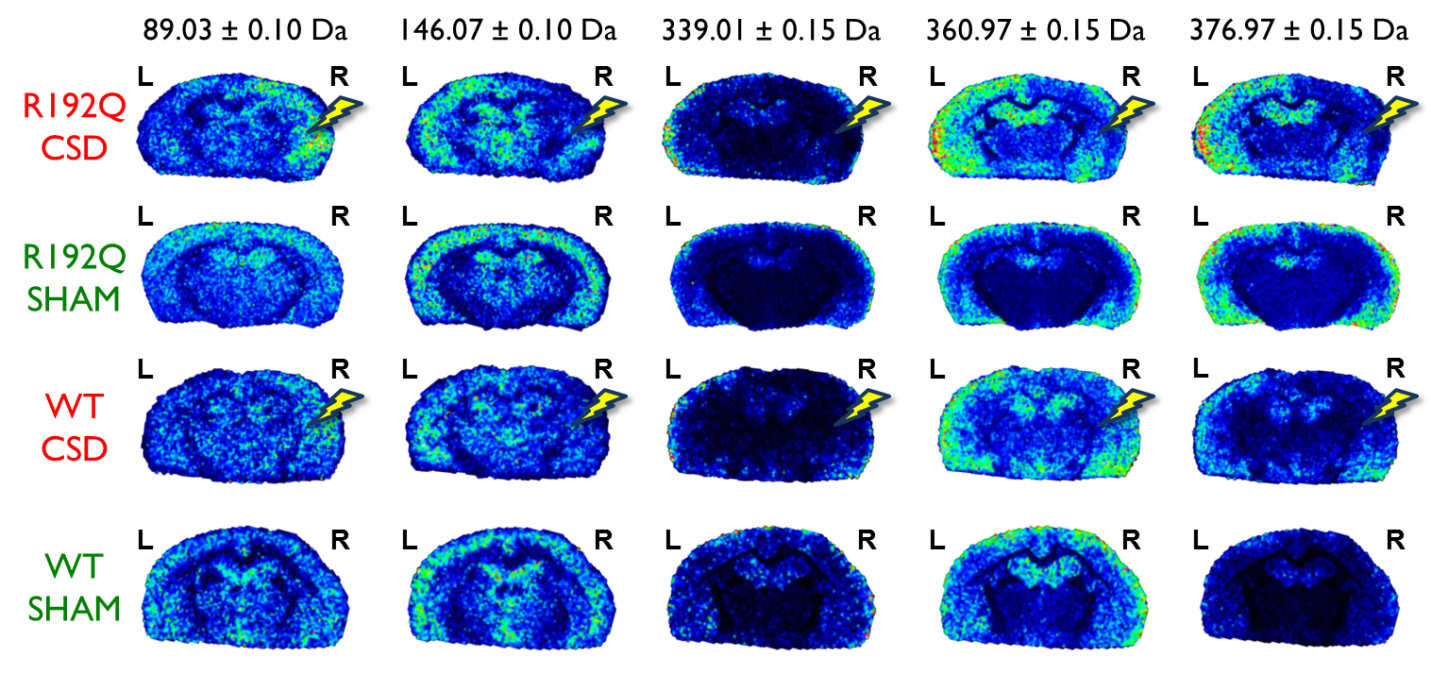
**

**Figure S2:** Representative spectra from different MSI datasets. **a)** Protein dataset – positive linear mode; 2000 – 20 000 Da. **b)** Peptide dataseet – positive reflectron mode; 600 – 2000 Da. **c)** Metabolite dataset – negative reflectron mode; 50 – 1000 Da.
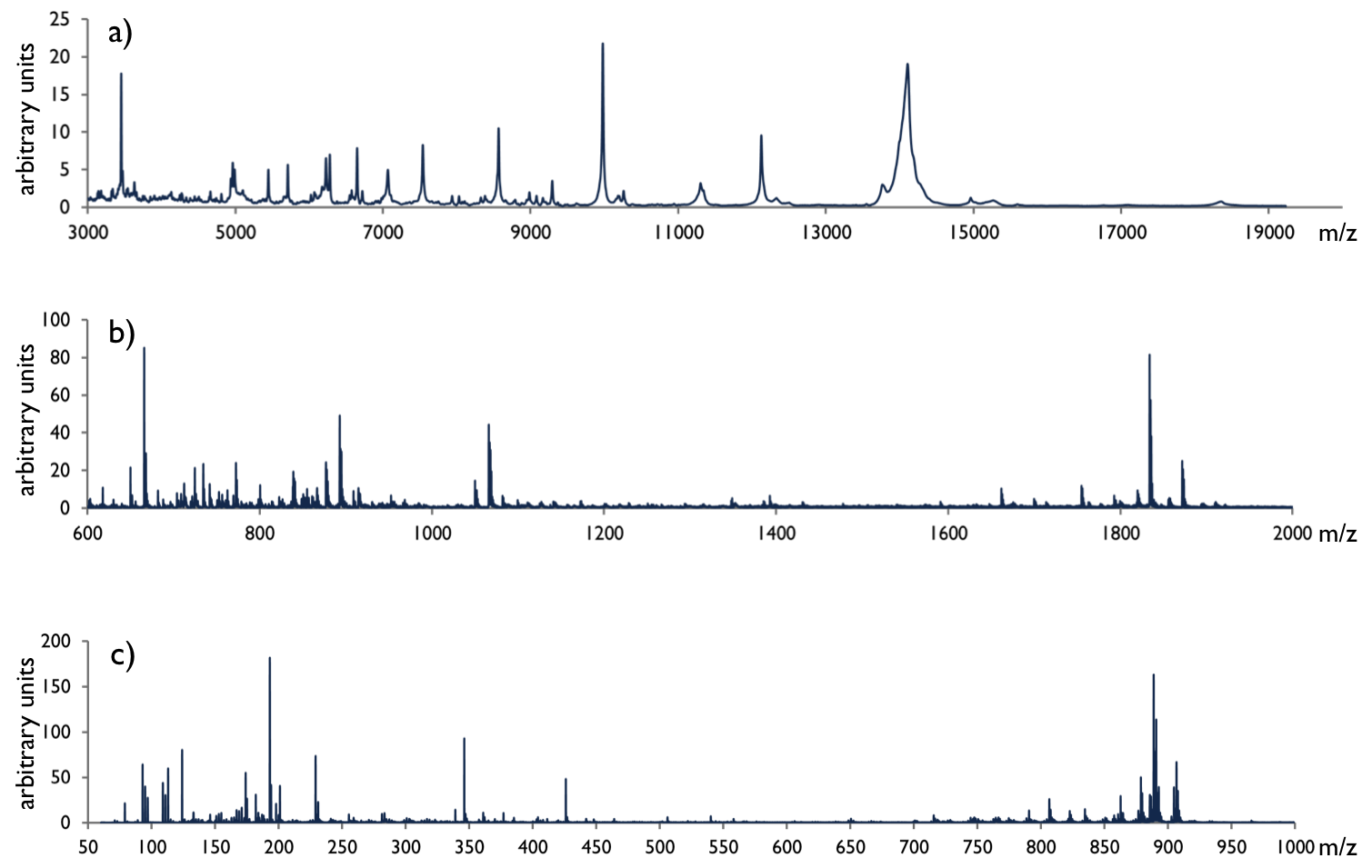

Supplement: Supplementary file 1 — (DOCX 1519 kb) [file 13361_2015_1136_MOESM1_ESM.docx]
